# Caries-resistant bonding layer in dentin

**DOI:** 10.1038/srep32740

**Published:** 2016-09-07

**Authors:** Wei Zhou, Li-na Niu, Lin Hu, Kai Jiao, Gang Chang, Li-juan Shen, Franklin R. Tay, Ji-hua Chen

**Affiliations:** 1 State Key Laboratory of Military Stomatology & National Clinical Research Center for Oral Diseases & Shaanxi Key Laboratory of Oral Diseases, Department of Prosthodontics, School of Stomatology, The Fourth Military Medical University, Xi’an, Shaanxi, China; 2 Department of Stomatology, Qinghe Clinic of PLA Rocket Force General Hospital, Beijing, China; 3 Department of Endodontics, The Dental College of Georgia, Augusta University, Augusta, Georgia, USA

## Abstract

The present study examined the mechanism for caries resistance and the pulp responses in vital teeth following the use of the augmented-pressure adhesive displacement technique. Dentin adhesives were applied to the surface of sound dentin disks in 4 experimental groups: non-antibacterial adhesive and gentle adhesive displacement (N-G), non-antibacterial adhesive and augmented-pressure adhesive displacement (N-H), antibacterial adhesive and gentle adhesive displacement (A-G), antibacterial adhesive and augmented-pressure adhesive displacement (A-H). The depth of demineralization induced by biological or chemical demineralization models was measured using confocal laser scanning microscopy and analyzed with two-way ANOVA. Pulp responses of vital dog’s teeth to the augmented-pressure adhesive displacement technique were evaluated using light microscopy. Depth of demineralization was significantly affected by “adhesive type” and “intensity of adhesive displacement” for biological demineralization. For chemical demineralization, only “intensity of adhesive displacement” showed significant influence on lesion depth. Pulp response of 0.1, 0.2 and 0.3 MPa groups showed only moderate disorganization of the odontoblast layer at 24 hours that completely re-organized after 3 weeks. Augmented-pressure adhesive displacement improves the caries resistance property of bonded dentin and does not cause irreversible pulpal damage to vital teeth when the air pressure employed is equal or smaller than 0.3 MPa.

Dentin adhesives and resin composites are widely used in restorative dentistry^1^. Despite intensive research and development in this arena, the clinical failure rate of methacrylate resin-based restorative materials remains unsatisfactory. Secondary caries is the major cause for replacement of resin-based restorations^2^,^3^. Compared to enamel, there is a more organic substance in dentin, predominantly collagen. For dentin bonding, incomplete infiltration of resin into demineralized dentin and hydrolysis of the polymerized resin result in exposure of the dentin collagen matrix. The activity of collagen-bound matrix metalloproteinases is increased by acid-etching, which promotes collagen degradation. The weakened bonding interface becomes a pathway for invasion of cariogenic microorganisms. Acids and enzymes produced by bacteria further deteriorate the adhesive polymer matrix and denature the denuded dentin collagen matrix^2^,^4^,^5^,^6^. Thus, development of clinically-relevant strategies for improving the durability of resin-dentin bonds and prevention of oral bacteria invasion is a high priority in adhesive dentistry.

The authors developed an augmented-pressure adhesive bonding technique with the use of 0.3 MPa air-spray pressure for adhesive displacement^7^. This dentin bonding technique enhances resin infiltration into the acid-etched dentin, which has potential to improve bond durability^7^. With the use of an *in vitro* artificial caries model, the bonding layer created by a commercial, non-antibacterial, 2-step etch-and-rinse adhesive using augmented-pressure adhesive displacement was resistant to the initiation of secondary caries along the bonding interface. This caries resistance effect was similar to the effect exhibited by the use of an experimental antibacterial adhesive, in the absence of augmented-pressure adhesive displacement, on protecting the bonding interface from artificial caries^8^. Although it is known that the anti-caries property of the antibacterial adhesive originates from the bactericidal effect of quaternary ammonium salts^9^,^10^,^11^,^12^, the mechanism responsible for the caries-resistance effect produced by the augmented-pressure adhesive displacement technique remains unclear.

Apart from caries-resistance, operational safety is another important issue which influences the clinical translation of this technique. Based on the hydrodynamic theory, application of strong air-blow to exposed dentin may induce rapid outward fluid flow from the dentinal tubules. This may result in the aspiration of odontoblasts into the dentinal tubules, which, in turn, may elicit a painful response by stimulation of the A-δ nerve fiber endings in the vicinity of the pulpodentinal junction^13^,^14^. In the application of the augmented-pressure adhesive displacement technique, exposed dentinal tubules are first filled with adhesive prior to the application of strong air-blow. Because the tubules are already filled with fairly viscous adhesive resin monomers, there should be minimal loss of dentinal fluid by convection and evaporation under strong air-blow^8^. Although this technique is theoretically unlikely to produce adverse reactions from the dental pulp, such a speculation requires further experiment support.

Accordingly, the objectives of the present study were to investigate the mechanism responsible for the caries resistance property of the augmented-pressure adhesive displacement technique, and to examine the pulpal responses of vital teeth bonded *in vivo* using this technique. The first hypothesis tested was that the caries resistance property of the augmented-pressure adhesive displacement technique is attributed to improvement of the integrity of the bonded interface instead of the antimicrobial activity of the adhesive. The second null hypothesis tested was that application of strong air-blow to exposed dentin using the augmented-pressure adhesive displacement technique does not result in irreversible damage to the dental pulp.

## Materials and Methods

### Specimen preparation

Twenty-four caries-free human third molars were collected after informed consent was obtained from the patients. The protocol employed was approved by the Ethics Committee for Human Studies of the Fourth Military Medical University. All the experiments were carried out in accordance with the approved guidelines and regulations.

The occlusal enamel of each tooth was removed with a horizontal cut using a slow-speed diamond saw (MTI Co., Shenyang, China) under water-cooling. A second parallel cut was made 2 mm beneath the first cut to obtain a dentin disk containing mid-coronal dentin. The occlusal surface of each dentin disk was polished with 600-grit silicon carbide abrasive paper under water-cooling for 30 sec to create a standardized smear layer^15^. Each tooth segment was attached with cyanoacrylate glue (Zapit, Dental Ventures of America, Anaheim Hills, CA, USA) to a Plexiglas platform assembly to deliver 20 cm of water pressure during bonding^16^. In all groups, the dentin surfaces were etched with 37% phosphoric etching gel (Scotchbond™ Universal Etchant, 3 M ESPE, St. Paul, MN, USA) for 15 sec, rinsed and dried gently for the next process. The adhesives used in the present study include a commercial 2-step etch-and-rinse adhesive (Single Bond 2, 3 M ESPE) and an experimental antibacterial adhesive. The latter was produced by adding 10 wt% 2-methacryloxylethyl dodecyl methyl ammonium bromide to Single Bond 2^11^.

Four groups were evaluated: non-antibacterial adhesive + gentle air-blowing (N-G), non-antibacterial adhesive + hard air-blowing (N-H), antibacterial adhesive + gentle air-blowing (A-G), antibacterial adhesive + hard air-blowing (A-H). For gentle air-blowing, the adhesive was thinned with 0.1 MPa air pressure blowing for 5 sec at a distance of 1 cm from the dentin surface. The air pressure used for high pressure air-blowing was 0.3 MPa. A custom-made switch and pressure gauge was used to control the output air pressure delivered from a triple syringe.

Each dentin disk was divided into two halves. Each half was treated with the same adhesive but using different air-blowing methods. A groove was made with the diamond saw in the middle of dentin disk. A plastic card (60 mm high × 90 mm wide × 1 mm thick) was inserted into the groove when air-blowing was performed so that bonding on one-half of the disk was not affected by bonding performed on the other half. After adhesive application, light curing was performed using a light-emitting unit (Spectrum^®^ 800, Dentsply, York, PA, USA; output intensity: 450 mW/cm^2^) for 20 sec. This split-tooth bonding method was used to reduce sampling error caused by the variance among the different teeth. Except for the air pressure and adhesive used, all procedures were the same for the four groups. Adjustment of the switch and pressure gauge was used to control the output air pressure of a triple syringe. Specimens were then stored in water for 24 h at 37 °C for further tests and observations. A flow chart of the experimental design is presented in Supplementary S1.

### Bacterial and chemical demineralization

#### Biofilm formation *in vitro*

*Streptococcus mutans* (ATCC 25175, American Type Culture Collection, Manassas, VA, USA) was used to create a single-species acidogenic bacterial biofilm for the biological demineralization. The *S. mutans* was maintained in brain heart infusion (BHI) broth (Hopebio, Qing Dao, China) and cultured anaerobically at 37 °C (5% CO_2_ by volume) for 24 h. The bacterial suspension was diluted to 10^7^ CFU/mL using BHI broth supplemented with 1% sucrose^17^.

Sixteen adhesive-bonded dentin disks (32 adhesive-bonded disk-halves) were used in this part of the study. The specimens were coated with nail varnish except for the adhesive-bonded surfaces. The specimens were sterilized by exposure to ultraviolet light for 4 h in a super clean bench^8^. Each specimen was then placed in one of the wells of a sterile 24-well plate. The *S. mutans* suspension was prepared freshly as described above and were added to each specimen-containing well. The plate was incubated in an anaerobic work station (Whitley H85 hypoxystation, Shipley, West Yorkshire, United Kingdom) for 30 days or 45 days (n = 4 for each designated time period per group), and the growth medium was refreshed every two days. The pH values of the bacterial suspensions were monitored using a pH meter (ORION 420A, Thermo Scientific Orion, Wayne, MI, USA) from the first day to the last day of the experimental period to confirm the vitality of the acidogenic bacteria biofilms growing on the surface of the bonded dentin disks.

#### Chemical demineralization

Eight adhesive-bonded dentin disks (16 adhesive-bonded disk-halves; n = 4 per group) were used in this part of the study. The specimens were coated with nail varnish except for the adhesive-bonded surfaces. The specimens were sterilized by exposure to ultraviolet light for 4 h and then incubated at 37 °C for 6 days in 10 mL of partially-saturated acidic buffer system (pH 4.3). The composition of partially-saturated acidic buffer system was 75 mmol/L glacial acetic acid, 2 mmol/L CaCl_2_, 2 mmol/L KH_2_PO_4_, 1 mmol/L NaN_3_ and 0.1 mmol/L NaF^18^,^19^,^20^.

#### Confocal laser scanning microscopy

The specimens subjected to bacterial demineralization were sonicated in distilled water to remove as much of the attached bacterial biofilms as possible before slicing. All the bonded dentin disks involved in bacterial and chemical demineralization were sectioned into slices in a direction parallel to the longitudinal axis of the tooth, using the slow-speed diamond saw under water cooling. Both sides of each slice were serially-polished with 400, 800, 1200 and 2000 grit silicon carbide papers and sonicated with distilled water. Four 0.5 mm thick slices were obtained from each dentin disk-halves. Each slice was placed on a microscopic glass slide, covered with ethylene glycol to prevent water evaporation^21^,^22^, and examined wet using confocal laser scanning microscopy (FV1000, Olympus Corp., Tokyo, Japan). The excitation wavelength was 488 nm and a 505–580 nm band-pass filter was used for detecting the autofluorescence emitted from the specimens^23^,^24^,^25^,^26^,^27^. The scanning thickness was 5 μm for all specimens. Images were obtained using FV10-ASW 3.1 Viewer (Olympus Corp., Tokyo, Japan). The depth of demineralization was measured from the surface to the lowest level of autofluorescence exhibited by the demineralized lesion. For each slice, three randomly selected sites were measured and the mean of the three data represent the mean lesion depth of each slice. For each group (N-G, N-H, A-G and A-H), the use of 4 dentin disks with 4 slices per disk and 3 sites of measurement per slice resulted in 48 measurements per group. Because the 4 slices were taken from the same tooth, the mean of the data obtained from the 4 slices of each dentin disk was used to represent the mean lesion depth of each disk. This resulted in 4 mean values in each group for statistical analysis.

#### Scanning electron microscopy and energy dispersive X-ray analysis

After examination with confocal laser scanning microscopy, each dentin slice was etched with 37% phosphoric acid for 5 sec to bring the polished surface into relief. After air-drying, the specimens were attached to aluminum stubs and sputter-coated with gold-palladium to observe the dentin in the longitudinal section using scanning electron microscopy (Hitachi FE-SEM 4800, Tokyo, Japan) at 5 kV. Demineralization of the dentin surfaces was evaluated by the energy dispersive X-ray analysis (EDS, PV72-45030LC, Ametek, USA) unit attached to the SEM, using an accelerating voltage of 30 kV. Map-scans were used to analyze the element percentage (wt%) of carbon (C), oxygen (O), silicon (Si), phosphorus (P) and calcium (Ca).

#### Statistical analysis

Because no obvious demineralization was detected from the N-H, A-G, and A-H groups after 30 days of bacterial challenge, only data obtained from the 45-day bacterial demineralization and 6-day chemical demineralization were used for statistical analysis. Two-factor analysis of variance (ANOVA) was used to separately analyze the results obtained from 45-day bacterial demineralization and 6-day chemical demineralization in the 4 groups. For each analysis, the effects of “adhesive type”, “intensity of adhesive displacement” and the interaction of those 2 factors on demineralization depth were analyzed. Post-doc pairwise comparisons were conducted using the Holm-Sidak statistic. Parametric testing methods were employed after ascertaining the normality and equal variance assumptions of the data sets. If the data did not satisfy those assumptions, the data sets were nonlinearly-transformed to satisfy the assumptions prior to the use of parametric statistical methods. For all analyses, statistical significance was pre-set at α = 0.05.

### Pulpal response to adhesive processed by different air-blowing method

Sixteen teeth, including 8 premolars and 8 molars derived from the maxillary and mandible of one 2-year old male beagle dog were used for this part of the experiment. The dog was obtained from the Animal Center of the Fourth Military Medical University and weighed 6 kg. All the procedures conducted on the beagle dog were performed in accordance with the guidelines and regulations for the care and use of laboratory animals of Fourth Military Medical University. The protocol was approved by the Animal Research Ethics Committee of School of Stomatology, Fourth Military Medical University, Xi’an, China.

Four groups were examined: Single Bond 2 with gentle air pressure displacement (air pressure 0.1 MPa) and Single Bond 2 with augmented air pressure displacement (air pressure 0.2, 0.3 and 0.4 MPa). Eight teeth from the left side of maxillary and mandible were assigned to the 4 groups randomly and treated accordingly using the bonding protocol described in the previous section. The same procedures were performed on the eight teeth from the right side of the maxillary and mandible 3 weeks later. Such a design enabled data to be collected on pulpal responses after 24 hours and after 3 weeks of restoration. The experimental antibacterial adhesive was not used because the objective of this part of the study was to examine the potential adverse effects of air pressure intensity associated with the augmented-pressure adhesive displacement technique on the dental pulp.

The animal was anesthetized by intraperitoneal injection of 2.5% pentobarbital sodium (Sigma-Aldrich Chemical Co., St. Louis, MO, USA, 35 mg/kg body wt.). All the teeth were isolated with rubber dam. The operative area around the mouth was disinfected with 1% tincture of iodine followed by 75% ethanol. The oral cavity was disinfected with 3% hydrogen peroxide. Class V cavities (4 mm × 3 mm × 1 mm) were prepared on the buccal surface of each tooth with tungsten carbide burs with water cooling. Each cavity was etched with 37% phosphoric acid gel (Scotchbond™ Universal Etchant) for 15 sec, bonded with the Single Bond 2. The adhesive was displaced using one of the four air pressure intensities as described in the group designations. Following adhesive displacement, the adhesive was light-cured for 20 sec. Each cavity was subsequently filled with resin composite (FiltekTM Z250, 3 M ESPE).

The dog was sacrificed after 24 hours when all the treatments were completed, using bilateral carotid artery perfusion with 4% paraformaldehyde. The maxillary and mandible were removed and each tooth was extracted from the bone. All the teeth were re-fixed by immersing in 4% paraformaldehyde solution for 2 weeks. After 10 weeks of demineralization with 10% EDTA (pH 7.4), all the teeth were rinsed with running water overnight, dehydrated in ascending grades of ethanol, cleared by xylol, and embedded with paraffin. The specimens were sectioned into 4 μm-thick slices. The slices were stained using hematoxylin-eosin and examined using light microscopy (BX-43, Olympus Corp., Tokyo, Japan)^28^,^29^,^30^.

## Results

The pH values of the bacterial solutions in the four groups were under 5. The values were maintained at the same level from the first day to the end of incubation (Supplementary S2).

**Figure 1:**
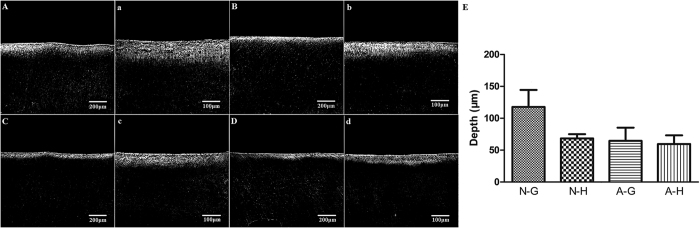
Confocal laser scanning microscopy images and quantitative results of dentin demineralization in the 4 experimental groups after 45 days of *S. mutans* challenge. (**A**) N-G group - 100X; (**a**) N-G group - 200X; (**B**) N-H group - 100X; (**b**) N-H group - 200X; (**C**) A-G group – 100X; (**c**) A-G group – 200X; (**D**) A-H group – 100X; (**d**) A-H group – 200X. DD: demineralized dentine. SD: sound dentin. (**E**) Bar chart showing variation in lesion depth (values represent means and standard deviations) in the 4 groups.

Confocal laser scanning microscopy images of bacterial demineralization generated along the restoration-dentin interface of the 4 groups after 30 days of bacterial challenge are shown in Supplementary S3. Only the N-G group showed slight dentin demineralization (Supplementary S3A,a). Demineralization was minimal and part of hybrid layer still existed in the other 3 groups (Supplementary S3B–D and b–d). In contrast with the 30-day results, extensive demineralization was identified along the restoration-dentin interface of the 4 groups after 45 days. The depth of demineralized dentin in the N-G group was the most extensive, while superficial dentin demineralization could be identified in the other three groups (Fig. 1A–D,a–d). Two-factor ANOVA indicated that the depth of demineralized dentin was significantly affected by the “intensity of adhesive displacement” (gentle *vs* augmented-pressure adhesive displacement; p < 0.001) and by “adhesive type” (non-antibacterial adhesive *vs* experimental antibacterial adhesive; p = 0.008). The interaction of these two factors was also statistically significant (p = 0.028). Quantitative results derived from the 4 groups are depicted in Fig. 1E. Within the factor “adhesive type”, significant difference was observed for pairwise comparison between gentle *vs* hard air-blowing when the non-antibacterial adhesive was employed (P = 0.004), while no significant difference was observed between gentle *vs* hard air-blowing when the antibacterial adhesive was used (P = 0.658). Within the factor “intensity of adhesive displacement”, significant difference was observed for pairwise comparison between the non-antibacterial *vs* antibacterial adhesive with the use of gentle air-blowing (P < 0.001), while no significant difference was observed between the non-antibacterial *vs* antibacterial adhesive with the use of hard air-blowing (P = 0.280).

**Figure 2:**
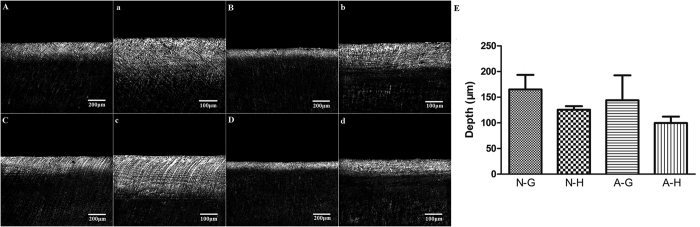
Confocal laser scanning microscopy images and quantitative results of dentin demineralization in the 4 experimental groups after 6 days of chemical demineralization. (**A**) N-G group - 100X; (**a**) N-G group - 200X; (**B**) N-H group - 100X; (**b**) N-H group - 200X; (**C**) A-G group – 100X; (**c**) A-G group – 200X; (**D**) A-H group – 100X; (**d**) A-H group – 200X. DD: demineralized dentine. SD: sound dentin. (**E**) Bar chart showing variation in lesion depth (values represent means and standard deviations) in the 4 groups.

Confocal laser scanning microscopy images of the restoration-dentin interface of the 4 groups after 6 days of chemical demineralization are depicted in Fig. 2(A–D,a-d). More extensive demineralization was observed in the 4 groups after 6 days of acetic acid treatment. Two-factor ANOVA indicated that the depth of demineralized dentin was significantly affected by the “intensity of adhesive displacement” (p = 0.006) but not by “adhesive type” (p = 0.107). The interaction of these two factors was not statistically significant (p = 0.831). Quantitative results derived from the 4 groups are depicted in Fig. 2E. Within the factor “adhesive type”, no significant difference was observed for pairwise comparison between gentle *vs* hard air-blowing when the non-antibacterial adhesive was employed (P = 0.057). Conversely, significant difference was observed between gentle *vs* hard air-blowing when the antibacterial adhesive was used (P = 0.018). Within the factor “intensity of adhesive displacement”, no significant difference was observed for pairwise comparison between the non-antibacterial *vs* antibacterial adhesive, irrespective of whether gentle air-blowing (P = 0.167) or hard air-blowing (P = 0.336) was adopted for adhesive displacement.

**Figure 3:**
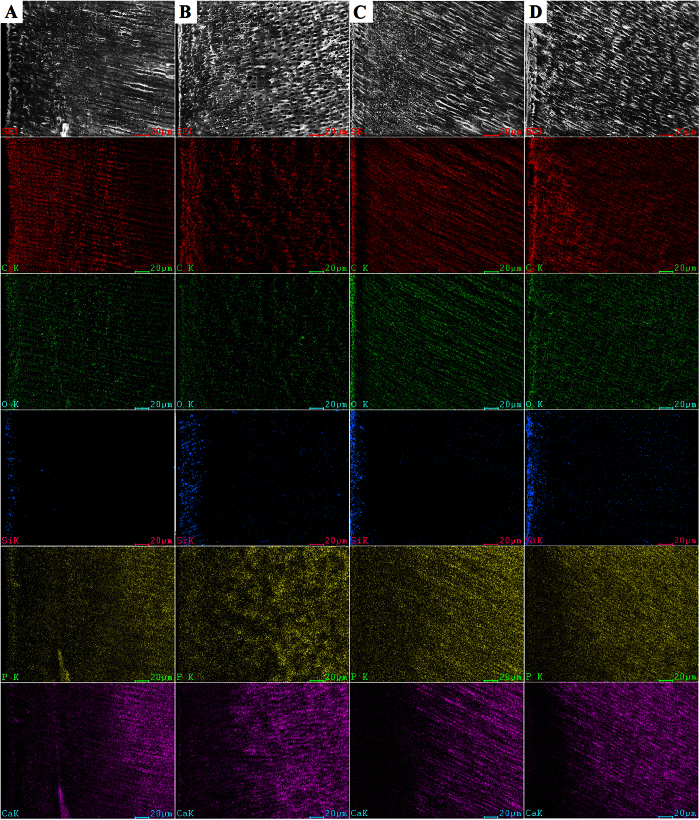
Scanning electron microscopy (500X) of the morphology of the resin-dentin interface and energy dispersive X-ray map-scans of the changes in elemental distribution from the surface to deep dentin induced by 45 days of *S. mutans* challenge in the four groups. (**A**) N-G group; (**B**) N-H group; (**C**) A-G group; (**D**) A-H group. C: carbon; O: oxygen; Si: silicon; Ca: calcium; P: phosphorus.

**Figure 4:**
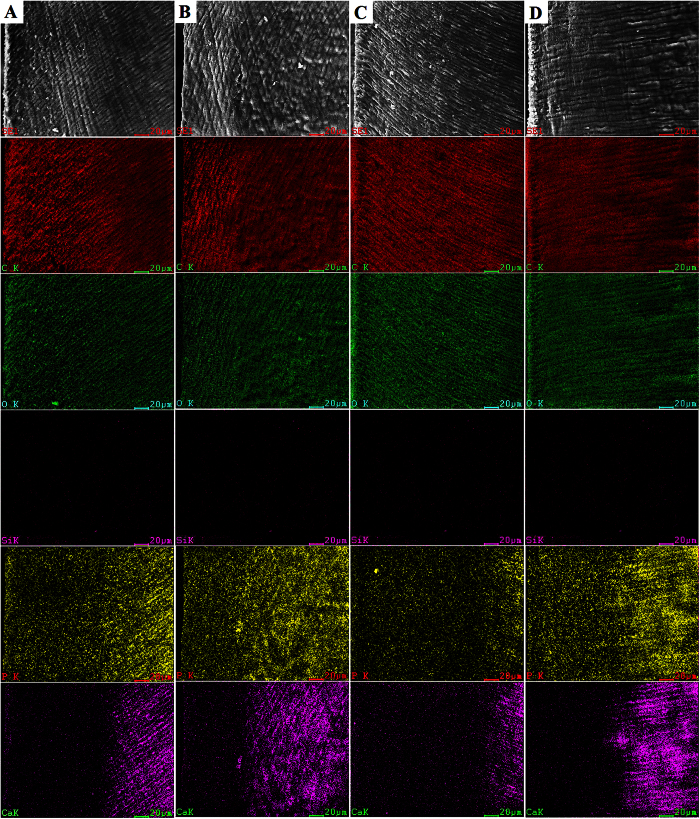
Scanning electron microscopy of the morphology of the resin-dentin interface and energy dispersive X-ray map-scans of the changes in elemental distribution from the surface to deep dentin induced by 6 days chemical caries challenge in four groups. (**A**) N-G group; (**B**) N-H group; (**C**) A-G group; (**D**) A-H group. C: carbon; O: oxygen; Si: silicon; Ca: calcium; P: phosphorus.

Scanning electron microscopy and EDS map-scans of 30-day bacterial demineralization are shown in Supplementary S4. Those representing 45-day bacterial demineralization and 6-day chemical demineralization are shown in Figs 3 and 4, respectively. Demineralized depths are represented by regions in the EDS map scans where there were lower Ca and P contents and higher C and O contents along the specimen surface. The tendency of demineralization shown by the EDS map scans was similar to that depicted by confocal laser scanning microscopy. Silicon is one of the component of Single Bond 2, which only could be clearly detected in the superficial dentin of specimens in the N-H, A-G and A-H groups after 30 days of bacterial demineralization (Supplementary S4B–D); only vague images of Si were identified from the N-G group after 30 days of bacterial demineralization (Supplementary S4A). Similarly, only vague images of Si could be identified from the N-H, A-G and A-H groups after 45 days of bacterial demineralization (Fig. 3B–D). By contract, the Si component of Single Bond 2 completely disappeared after 6 days of chemical demineralization in acetic acid (Fig. 4A–D).

**Figure 5:**
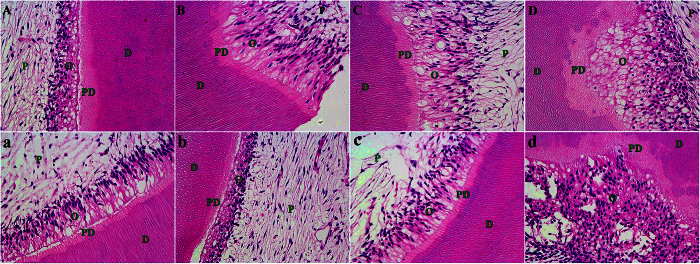
Representative images (400X) of pulp responses to different air pressure adhesive displacement. (**A**) 0.1 MPa after 24 hours (**a**) 0.1 MPa after 3 weeks; (**B**) 0.2 MPa after 24 hours; (**b**) 0.2 MPa after 3 weeks; (**C**) 0.3 MPa after 24 hours; (**c**) 0.3 MPa after 3 weeks; (**D**) 0.4 MPa after 24 hours; (**d**) 0.4 MPa after 3 weeks. D: dentin. PD: predentin. O: odontoblast layer. P: pulp.

Pulp responses to the displacement of adhesives with gentle and augmented-pressure adhesive displacement are shown in Fig. 5. For the 24-hour observation period, the 0.1, 0.2 and 0.3 MPa air pressure groups revealed moderate disorganization of the odontoblast layer and the conditions were similar in these groups (Fig. 5A–C). The tissue disorganization was recovered much after 3 weeks in the 0.1, 0.2 and 0.3 MPa air pressure groups (Fig. 5a–c), which was similar to the normal dental pulp morphology. No pulpal inflammation was observed after 3 weeks. Conversely, severe tissue disorganization and pulpal inflammation were observed in the 0.4 MPa air pressure group (Fig. 5D). In addition, the adverse pulpal response did not recover after 3 weeks (Fig. 5d). No aspiration of odontoblasts into the dentinal tubules could be detected in all groups.

## Discussion

Two artificial demineralization models (biological and chemical) and two dentin adhesives (non-antibacterial adhesive Single Bond 2 and experimental antibacterial adhesive) were used in the present study to determine whether caries resistance was attributed to enhanced adhesive displacement into acid-etched dentin or the antibacterial property of the adhesive. *Streptococcus mutans* was used for biological demineralization challenge because of its role in the etiology of dental caries. Being a strong acid producer, this bacterial species readily induces tooth demineralization^31^,^32^,^33^. The partially-saturated acidic buffer solution adopted for the chemical demineralization model has been used in previous studies to produce artificial lesions simulating dental caries in a sterile environment^18^,^20^,^34^. The experimental antibacterial adhesive containing quaternary ammonium salts did not inhibit dentin demineralization in the chemical demineralization model. Should the augmented-pressure adhesive displacement technique show improved caries resistance in both the biological and chemical caries models, the effect is more likely to be attributed to enhanced adhesive displacement into the demineralized collagen matrix rather than the antimicrobial properties of the adhesive. In the present study, the anti-demineralization effect of the experimental antibacterial adhesive manifested in the biological caries model could no longer be observed when bonded specimens were challenged with the chemical caries model. These results enabled us to validate the first null hypothesis that the caries resistance property of the augmented-pressure adhesive displacement technique is attributed to improvement of the integrity of the bonded interface instead of the antimicrobial activity of the adhesive.

The pulp responses elicited by air-blowing revealed that moderate disorganization of that odontoblast layer which recovered after 3 weeks. This observation was identified in the 0.1, 0.2 and 0.3 MPa air pressure groups. Thus, within the limits of using air-blowing pressure that is ≤0.3 MPa, the second null hypothesis that application of strong air-blow to exposed dentin using the augmented-pressure adhesive displacement technique does not result in irreversible damage to the dental pulp cannot be rejected.

Demineralized lesion depth was measured by two methods including confocal laser scanning microscopy and scanning electron microscopy-EDS analysis. For confocal laser scanning microscopy, evaluation of dentin demineralization is based on the autofluorescence emitted from dental materials and dentin. Autofluorescence is common in biological tissues and has diagnostic applications. It is a kind of fluorescence emitted by endogenous fluorophores in biological specimens but not by the exogenous fluorescent dyes or genetically-engineered fluorophores^35^. The intensity of autofluorescence from demineralized dentin is much stronger than mineralized dentin under the same excitation wavelength in both the bacterial and chemical artificial caries model. Many studies indicate that there is a significant correlation between increase in autofluorescence and dentin caries. Acid demineralization was thought to be the major reason for the increase in autofluorescence in natural dentinal caries^23^,^24^,^25^,^36^,^37^. Scanning electron microscopy-EDS analysis was regarded as a reliable method for evaluation of the mineral loss in caries or artificial carious lesions; the lower percentages of Ca and P being the predominant feature in these natural and artificial lesions^38^,^39^. Hence, this combined morphological-elemental analytical technique was adopted to confirm the results of autofluorescence detection by confocal laser scanning microscopy.

Dentin demineralization usually begins at pH 6.7^40^. In the present study, the pH value of the bacterial suspensions decreased to 5 in the first day and remained steady in the four groups up to the end of the experimental period. This demonstrates that refreshing the bacteria growth medium every two days can maintain bacterial viability. The pH result also indicates that acidity of the bacterial suspension was low enough for demineralization of dentin. Both the experimental antibacterial adhesive and the augmented-pressure adhesive displacement technique were capable of enhancing the resistance to bacterial challenge. In the 30-day confocal laser scanning microcopy images, dentin demineralization was only observed in the N-G group (Supplementary S3A,a). The other three groups showed only a thin bright line on the dentin surface (Supplementary S3B–D). According to the results of the present study, this bright line with strong autofluorescence should represent the hybrid layer. The intense autofluorescence emitted from the hybrid layer is attributed to the loss of carbonated apatite crystallites from mineralized dentin and exposure of the collagen matrix by acid-etching^8^. After demineralization by *S. mutans*, the hybrid layer became discontinuous in the N-H, A-G and A-H groups (Supplementary S3b–d). However, the damage did not extend across the bonding interface and no demineralization was detected beneath the hybrid layer. In the 45-day bacterial demineralization model, demineralization of dentin was evident in all groups, with the N-G group exhibiting the most extensive demineralization (Fig. 1E). Dentin demineralization progressed rapidly during the additional 15 days of demineralization (compared with the 30-day model). Although both the experimental antibacterial adhesive and the augmented-pressure adhesive displacement technique could not completely protect the bonded dentin from demineralization by *S. mutans*, both strategies were able to decrease the degree of dentin demineralization under the same *in vitro* cariogenic condition.

A different scenario was observed in the 6-day chemical demineralization model. The demineralization protection effect of the experimental antibacterial adhesive was completely nullified. By contrast, specimens bonded using the augmented-pressure adhesive displacement technique was still resistant to demineralization by acetic acid (Fig. 2B,E). The functional ingredient of the experimental antibacterial adhesive is the quaternary ammonium salt 2-methacryloxylethyl dodecylmethyl ammonium bromide. This functional methacrylate resin monomer has been shown to inhibit *S. mutans* proliferation by modulating *gtfB* gene expression and disrupting its membrane integrity^11^,^41^. The antibacterial resin monomer cannot manifest any anti-demineralization effect in a sterile environment. On the contrary, the more structurally-intact adhesive-dentin layer produced by the augmented-pressure adhesive displacement technique appears to provide a more reliable physical barrier against the penetration of bacterial acids or acetic acid into dentin.

A critical issue examined in the present work is whether the augmented-pressure adhesive displacement technique can be used clinically without adverse effects on the dental pulpal in vital teeth. According to the hydrodynamic theory, strong air blasts applied to vital tooth cavities result in evaporation of the water component of the dentinal fluid^14^,^42^. The negative capillary pressure created causes rapid outward fluid flow and aspiration of odontoblast cell bodies into the dentinal tubules. This undesirable pulpal response may provoke pain and induce necrosis of the dental pulp^13^,^43^. In the present study, the histopathological images clearly showed that the augmented-pressure adhesive displacement technique did not cause displacement of odontoblasts into the dentinal tubules even when a very strong pressure was employed (0.4 MPa) that resulted in irreversible pulpal inflammation. This is because the dentinal tubules were already filled with the adhesive prior to the application of strong air-blowing. Because the orifices of the dentinal tubules were sealed by the adhesives, only the adhesive solvent could be removed under air-blowing^8^. The pulpal response results provided the necessary *in vivo* evidence to support our claim that the use of the augmented-pressure adhesive displacement technique does not generate a strong negative capillary pressure that causes displacement of odontoblast cell bodies into the dentinal tubules. It is important to highlight that tooth preparation and bonding treatment can induce transient pulp inflammation^29^,^44^,^45^. Findings of the present work are in agreement with those studies. The 0.1, 0.2 and 0.3 MPa air pressure groups produced similar pulpal responses showing reversible disorganization of the odontoblast layer at the pulpal-dentinal junction. The results confirm that displacing dentin adhesives with the use of 0.3 MPa air pressure is as safe as the widely-use clinical technique of displacing adhesive applied to a tooth cavity with 0.1 MPa air pressure delivered via the triple syringe of a dental unit.

## Conclusions

A more structurally intact adhesive-dentin mixed layer created by augmented-pressure adhesive displacement contributes to the caries-resistance effect of the bonding layer in dentin. This layer functions as a physical barrier that protects dentin from the damage of harmful biological and chemical challenges. Additionally, the pulp responses elicited by hard (equal or lower than 0.3 MPa) and gentle air-blowing are similar. These results attest the clinical feasibility of the augmented-pressure adhesive displacement technique in the application of dentin adhesives to acid-etched dentin.

## Additional Information

**How to cite this article**: Zhou, W. *et al*. Caries-resistant bonding layer in dentin. *Sci. Rep*. **6**, 32740; doi: 10.1038/srep32740 (2016).

## Supplementary Material

Supplementary Information
