# Supplementary material for: Caries-resistant bonding layer in dentin
Source: Sci Rep. 2016 Sep 7;6:32740. doi: 10.1038/srep32740 (PMC5013435; doi:10.1038/srep32740)
Supplement: Supplementary Information [file srep32740-s1.pdf]

Supplementary Information

Caries-resistant bonding layer in dentin

Wei Zhou[a,#], Li-na Niu[a, #], Lin Hu[b,#], Kai Jiao[a], Gang Chang[a], Li-juan Shen[a], Franklin R. Tay[c,]*, Ji-hua Chen[a,]*

[#:] Equal contributors

Table of Content

S1 A flow chart of the experimental design of the present study

S2 pH values of the bacterial solutions in the 4 experimental groups from 0 – 45 days

S3 Confocal laser scanning microscopy of dentin demineralization in the 4 groups after 30 days of *Streptococcus mutans* challenge

S4 Scanning electron microscopy of the morphology of the resin-dentin interface and energy dispersive X-ray map-scans of the elemental distribution from the surface to deep dentin in the 4 groups after 30 days of *Streptococcus mutans* challenge.

Supplementary S1

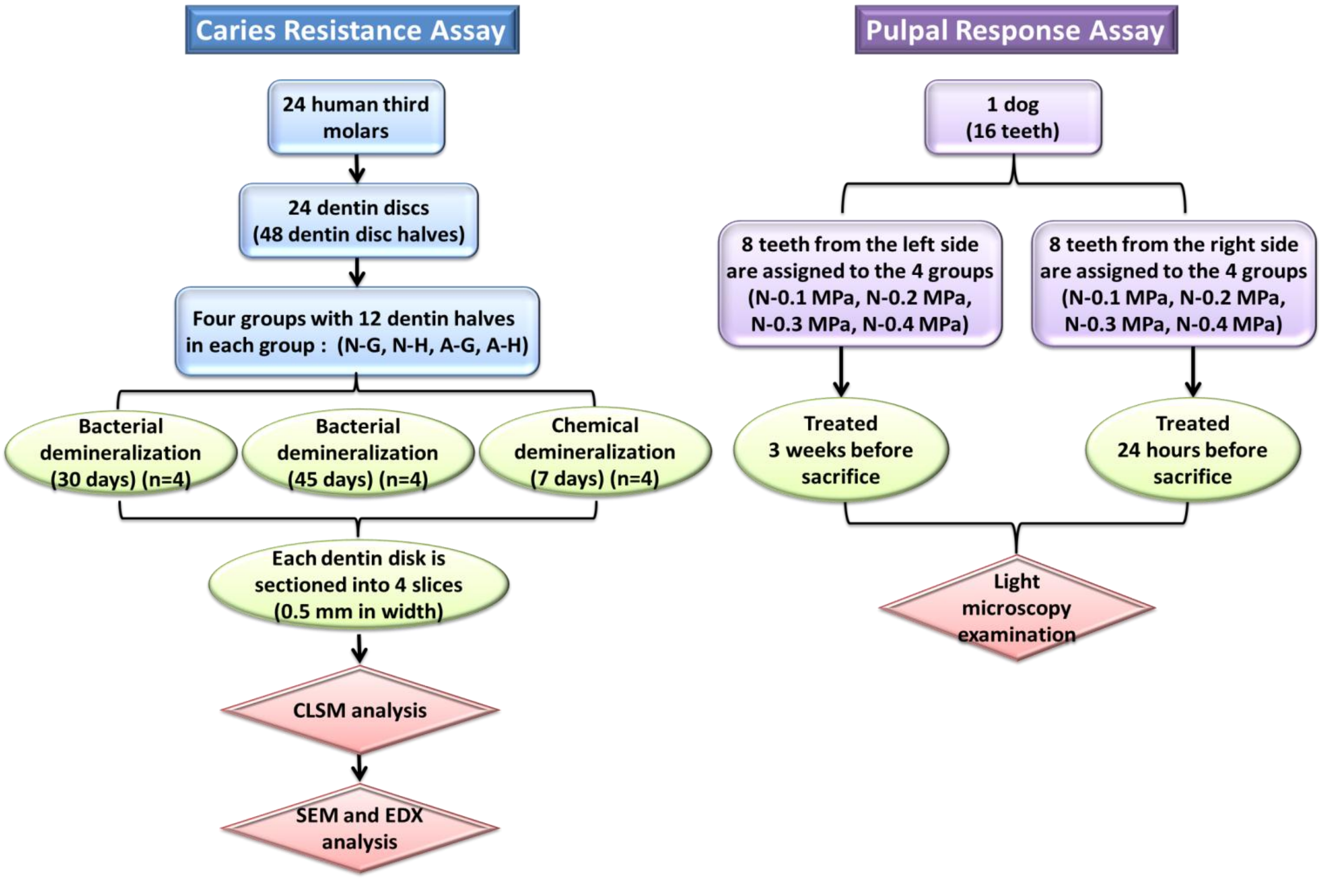

Figure S1 A flow chart of the experimental design of the present study
N-G: non-antibacterial adhesive + gentle air-blowing
N-H: non-antibacterial adhesive + hard air-blowing
A-G: antibacterial adhesive + gentle air-blowing
A-H: antibacterial adhesive + hard air-blowing
N-0.1 MPa: Single Bond 2 + gentle air-blowing (0.1 MPa)
N-0.2 MPa: Single Bond 2 + hard air-blowing (0.2 MPa)
N-0.3 MPa: Single Bond 2 + hard air-blowing (0.3 MPa)
N-0.4 MPa: Single Bond 2 + hard air-blowing (0.4 MPa)

Supplementary S2

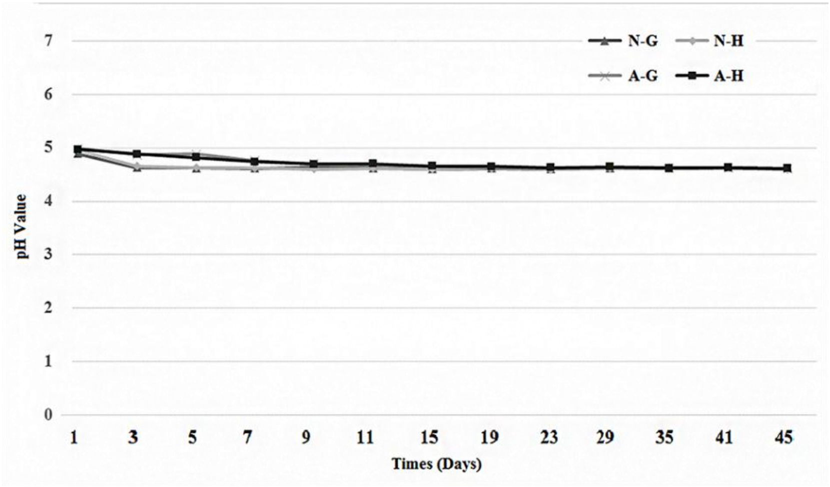

Figure S2 pH values of the bacterial solutions in the 4 experimental groups from 0 – 45 days.

Supplementary S3

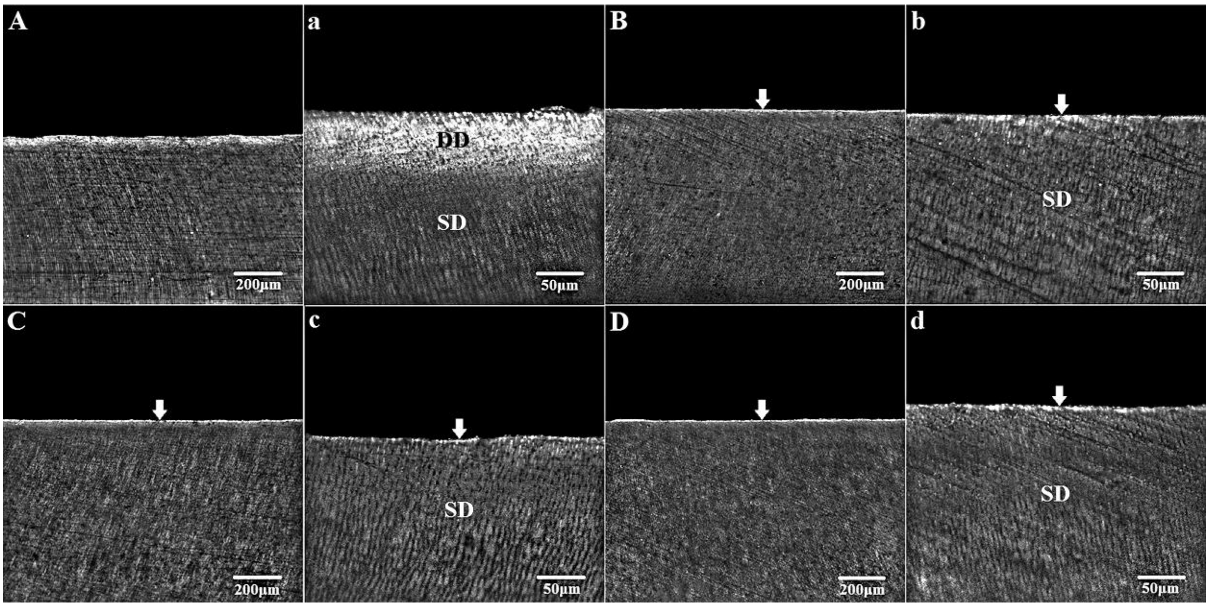

Figure S3 Confocal laser scanning microscopy of dentin demineralization in the 4 groups after 30 days of *Streptococcus mutans* challenge. (A) N-G group – 100X; (a) N-G group – 400X; (B) N-H group – 100X; (b) N-H group – 400X; (C) A-G group – 100X; (c) A-G group – 400X; (D) A-H group – 100X; (d) A-H group – 400X. Demineralized dentin (DD) emitted stronger autofluorescence than sound mineralized dentin (SD).

Supplementary S4

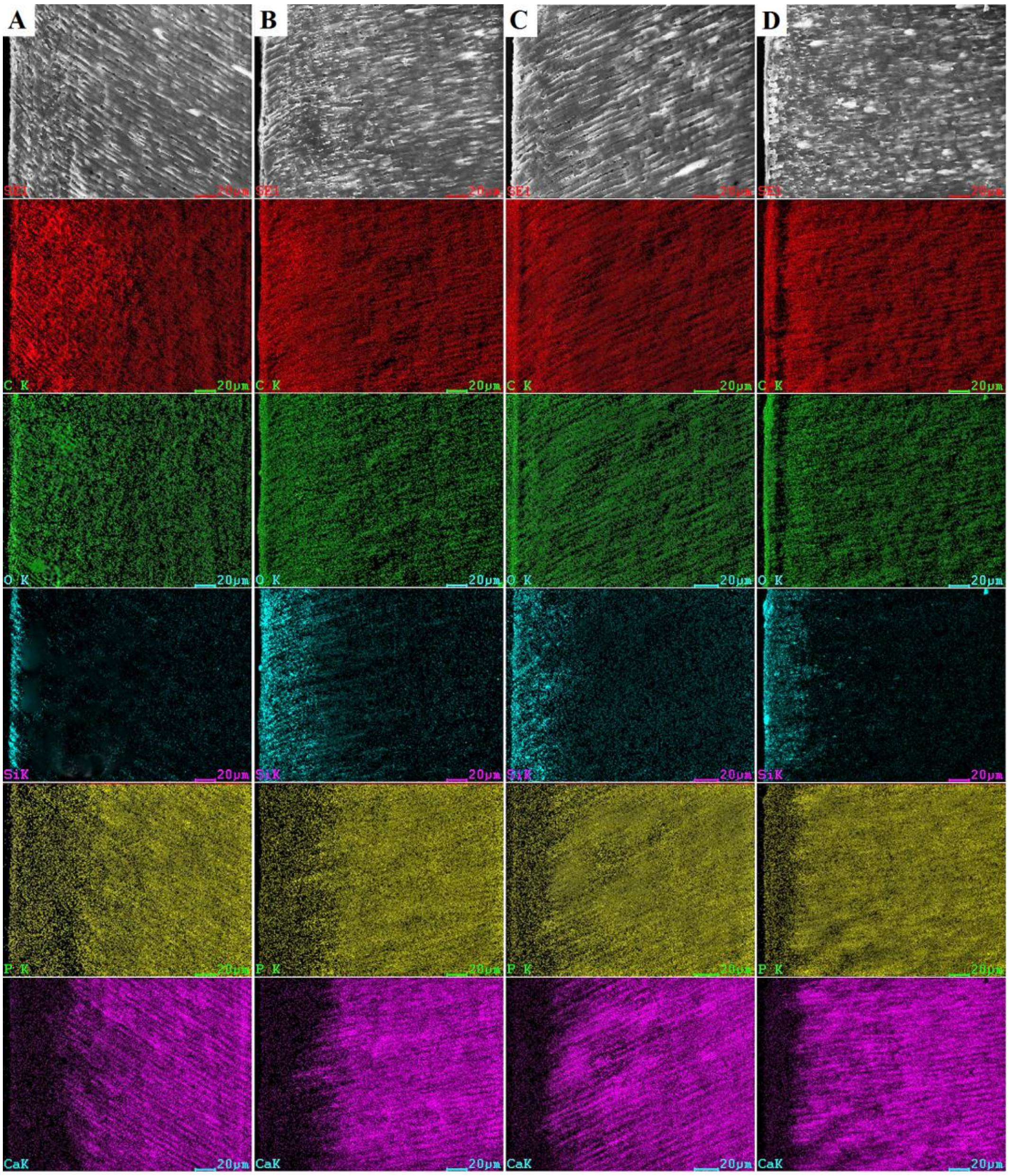

Figure S4 Scanning electron microscopy (500X) of the morphology of the resin-dentin interface and energy dispersive X-ray map-scans of the elemental distribution from the surface to deep dentin in the 4 groups after 30 days of *Streptococcus mutans* challenge. (A) N-G group; (B) N-H group; (C) A-G group; (D) A-H group. C: carbon; O: oxygen; Si: silicon; Ca: calcium; P: phosphorus.
